# Supplementary material for: Retroviral Infection and Commensal Bacteria Dependently Alter the Metabolomic Profile in a Sterile Organ
Source: Viruses. 2023 Jan 29;15(2):386. doi: 10.3390/v15020386 (PMC9967258; doi:10.3390/v15020386)
Supplement: Supplementary file 1 [file viruses-15-00386-s001.zip › Table S1.pdf]

Table S1: Known metabolites in the sera under positive ionization. Metabolites identified in the sera of GF, GF infected, *L. murinus* colonized, and *L. murinus* colonized and infected mice under positive ionization.

5-Methylcytidine	Creatine	Isoleucine	Creatinine	Leucine	Acetylcarnitine	Cystathionine	Linoleoylcarnitine	Alanine
Cytidine	Lysine	Methionine	Nicotinamide	Riboflavin	Betaine	Glu-Thr	2'-Deoxycytidine	Carnitine
3-Methylcytidine	Choline [M]+	homocitrulline	Taurine	Hypotaurine	Pipecolic acid	Phenylalanine	Urea	Arginine
Valine	Ornithine	Proline	Citrulline	Oleoyl-L-carnitine	Serine	N,N-Dimethylarginine	Guanidinoacetic acid	Palmitoylcarnitine
7,8-Dihydrobiopterin	gamma-Glutamylleucine	N6,N6-dimethyllysine	1-Methyladenosine	N-epsilon-Acetyllysine	N-.alpha.-Acetyl-L-arginine	Palmitoyl sphingomyelin	Myristoyl-L-carnitine	Methioninesulfoxide [M+H]+
2-Arachidonoylglycerol	Nalpha-acetyl-L-Lysine	SN-Glycero-3-Phosphocoline	1-Palmitoyl-sn-glycero-3-phosphocholine late	5-Methyl-2'-deoxycytidine	N-Oleoyl-D-erythro-sphingosylphosphorylcholine	Palmitoyleicosapentaenoyl phosphatidylcholine	1-Stearoyl-2-hydroxy-sn-glycero-3-phosphocholine	1-Heptadecanoyl-sn-glycero-3-phosphocholine late
1,2-Dilinoleoyl-sn-glycero-3-phosphocholine	1-(1Z-Octadecenyl)-2-(5Z,8Z,11Z,14Z-eicosatetraenoyl)-sn-glycero-3-phosphocholine	1-Oleoyl-sn-glycero-3-phosphoethanolamine	1-(1Z-Octadecenyl)-2-(4Z,7Z,10Z,13Z,16Z,19Z-docosahexaenoyl)-sn-glycero-3-phosphocholine	1-O-Hexadecyl-2-O-(5Z,8Z,11Z,14Z,17Z-eicosapentaenoyl)-sn-glyceryl-3-phosphorylcholine	1-(1Z-Hexadecenyl)-sn-glycero-3-phosphocholine late	1-O-Hexadecyl-2-O-(4Z,7Z,10Z,13Z,16Z,19Z-docosahexaenoyl)-sn-glyceryl-3-phosphorylcholine	1-Stearoyl-2-hydroxy-sn-glycero-3-phosphoethanolamine	1-Hexadecyl-2-(5Z,8Z,11Z,14Z-eicosatetraenoyl)-sn-glycero-3-phosphocholine
1,2-dioleoyl-sn-glycero-3-phosphatidylcholine	1-(1Z-Octadecenyl)-sn-glycero-3-phosphocholine early	1-Palmitoyl-2-docosahexaenoyl-sn-glycero-3-phosphocholine	1-(1Z-Hexadecenyl)-sn-glycero-3-phosphocholine early	1-Myristoyl-sn-glycero-3-phosphocholine	1-O-Octadecyl-sn-glyceryl-3-phosphorylcholine	1-O-Hexadecyl-2-O-acetyl-sn-glyceryl-3-phosphorylcholine	1-(1Z-Octadecenyl)-sn-glycero-3-phosphocholine late	1-Palmitoyl-sn-glycero-3-phosphocholine early
1,2-Dipalmitoleoyl-sn-glycero-3-phosphocholine								
