# Supplementary material for: Retroviral Infection and Commensal Bacteria Dependently Alter the Metabolomic Profile in a Sterile Organ
Source: Viruses. 2023 Jan 29;15(2):386. doi: 10.3390/v15020386 (PMC9967258; doi:10.3390/v15020386)
Supplement: Supplementary file 1 [file viruses-15-00386-s001.zip › Table S2.pdf]

Table S2: Metabolites identified in the sera under negative ionization. Known metabolites in the sera of GF, GF infected, *L. murinus* colonized, and *L. murinus* colonized plus infected mice under negative ionization.

Citrulline	4-Nitrophenol	Cytidine	Allantoin	Hypotaurine	Arabitol	Isoleucine	Adipic acid
Arginine	Leucine	Asparagine	lysoPC 14:0	Citric acid	Vanillin	Thymidine	Taurine
Serine	Pseudouridine	myo-Inositol	lysoPC 16:0 early	lysoPC 18:2 late	lysoPC 18:0 late	lysoPE 16:0 late	lysoPC 18:2 early
Delta-Hydroxylysine	lysoPC 18:0 early	lysoPE 16:0 early	lysoPC 17:0 late	lysoPC 20:4 late	lysoPC 16:0 late	lysoPC 20:4 early	N-Acetylalanine
lysoPC 18:1 late	lysoPE 18:0 late	lysoPC 18:1 early	lysoPE 18:0 early	Pantothenic acid	Methioninesulfoxide	N-epsilon-Acetyllysine	N-Acetylleucine
Palmitoyl sphingomyelin	N-Acetylhistidine	N,N-Dimethylarginine	N-Methylglutamic acid	N-.alpha.-Acetyl-L-ornithine	Methyl-beta-galactopyranoside	2-Deoxyuridine	2-Mercaptobenzothiazole
2-Hydroxypalmitic acid	Beta.-Glycerophosphate	3-Hydroxyoctanoic acid	1-Palmitoyl-2-linoleoyl-sn-glycero-3-phosphocholine	1-Palmitoyl-2-docosahexaenoyl-sn-glycero-3-phosphocholine	1-Palmitoyl-2-arachidonoyl-sn-glycero-3-phosphocholine	1-(1Z-Hexadecenyl)-sn-glycero-3-phosphocholine	
