# Supplementary material for: Retroviral Infection and Commensal Bacteria Dependently Alter the Metabolomic Profile in a Sterile Organ
Source: Viruses. 2023 Jan 29;15(2):386. doi: 10.3390/v15020386 (PMC9967258; doi:10.3390/v15020386)
Supplement: Supplementary file 1 [file viruses-15-00386-s001.zip › Table S3.pdf]

Table S3: Known metabolites found in spleens under positive ionization. Metabolites identified in the spleens of GF, GF infected, *L. murinus* colonized, and *L. murinus* colonized and infected mice under positive ionization.

Nicotinamide	Ethanolamine	Anserine	Tyrosine	Pyridoxal	Threonine	Valine	Carnitine
Hypoxanthine	Pyrazinamide	Taurine	Urea	Tryptophan	Cytidine	Arginine	Phenylalanine
Aspartic acid	Methionine	Uracil	Xanthine	Choline [M]+	Serine	Proline	Alanine
Glutamic acid	Asparagine	Histidine	Thiamine Cation	Argininosuccinic acid	Sphinganine	Glucose-6-phosphate	N-epsilon-Acetyllysine
2'-Deoxycytidine	N,N-Dimethylarginine	Ergothioneine	3'-O-Methylinosine	Epsilon.-Caprolactam	N-Acetyl-D-glucosamine	Palmitoyl sphingomyelin	Nicotinamide riboside cation
N.epsilon.-Methyl-L-lysine	Gamma.-Aminobutyric acid	Methioninesulfoxide [M+H]+	1,2-Cyclohexanedione	1,2-Dilinoleoyl-sn-glycero-3-phosphocholine	1-Myristoyl-2-stearoyl-sn-glycero-3-phosphocholine	(2R)-3-Hydroxyisovaleroylcarnitine	1,2-Diarachidonoyl-sn-glycero-3-phosphocholine
1-O-Hexadecyl-2-O-(4Z,7Z,10Z,13Z,16Z,19Z-docosahexaenoyl)-sn-glyceryl-3-phosphorylcholine	1-(1Z-Octadecenyl)-2-(5Z,8Z,11Z,14Z-eicosatetraenoyl)-sn-glycero-3-phosphoethanolamine	1-(1Z-Octadecenyl)-2-(4Z,7Z,10Z,13Z,16Z,19Z-docosahexaenoyl)-sn-glycero-3-phosphoethanolamine	2-Docosahexaenoyl-1-stearoyl-sn-glycero-3-phosphoethanolamine	1-Stearoyl-2-linoleoyl-sn-glycero-3-phospho-L-serine	1,2-Dipentadecanoyl-sn-glycero-3-phosphocholine	2-Arachidonoyl-1-palmitoyl-sn-glycero-3-phosphoethanolamine	1,2-dioleoyl-sn-glycero-3-phosphatidylcholine
1-Palmitoyl-2-docosahexaenoyl-sn-glycero-3-phosphocholine	1-Oleoyl-2-myristoyl-sn-glycero-3-phosphocholine	2-Oleoyl-1-palmitoyl-sn-glycero-3-phosphocholine	Nepsilon, Nepsilon-Trimethyllysine				
