# Supplementary material for: Retroviral Infection and Commensal Bacteria Dependently Alter the Metabolomic Profile in a Sterile Organ
Source: Viruses. 2023 Jan 29;15(2):386. doi: 10.3390/v15020386 (PMC9967258; doi:10.3390/v15020386)
Supplement: Supplementary file 1 [file viruses-15-00386-s001.zip › Table S4.pdf]

Table S4: Identified metabolites found in spleens under negative ionization. Known metabolites identified in the spleens of GF, GF infected, *L. murinus* colonized, and *L. murinus* colonized and infected mice under negative ionization.

PE 38:4	Galactitol	Uracil	Xanthine	Arginine	Gly-Asp	Taurine	myo-Inositol
Threonine	Hypoxanthine	N-Acetylserine	2'-Deoxycytidine	Xylitol	N-Acetylalanine	2-Deoxyuridine	Allantoin
Aspartic acid	Glucose	PC 32:0	Serine	Threonic acid	PE 38:6	PC 32:1	Glutamine
D-Asparagine	Histidine	Gly-Gly	PS 36:2	PE 36:4	Valine	Orotic acid	Glutamic acid
Ribose-5-phosphate	Tyrosine	Cytidine	Pseudouridine	N-epsilon-Acetyllysine	Glucose-1-phosphate	Dihydroxyacetone phosphate	Glucose-6-phosphate
N,N-Dimethylarginine	Pyroglutamic acid	Palmitoyl sphingomyelin	Ribose-1-phosphate	Plasmenyl-PE 38:6	L-.gamma.-Glutamyl-L-glutamic acid	Plasmenyl-PE 36:4	Plasmenyl-PE 38:4
O-Phosphocolamine	2-Docosahexaenoyl-1-stearoyl-sn-glycero-3-phosphoserine	1-Stearoyl-2-arachidonoyl-sn-glycero-3-phosphoserine	1-Palmitoyl-2-docosahexaenoyl-sn-glycero-3-phosphocholine				
